# Genetic polymorphisms in the angiotensin converting enzyme, actinin 3 and paraoxonase 1 genes in women with diabetes and hypertension

**DOI:** 10.20945/2359-4292-2021-0204

**Published:** 2023-11-10

**Authors:** Gabrielle Gaspar Arejano, Laura Vargas Hoffmann, Linoska Ferreira Wyse, Poliana Espíndola Correia, Simone Pieniz, Fabiana Torma Botelho, Augusto Schneider, Ines Schadock, Carlos Castilho Barros

**Affiliations:** 1 Universidade Federal de Pelotas Laboratório de Nutrigenômica Pelotas RS Brasil Laboratório de Nutrigenômica, Universidade Federal de Pelotas, Pelotas, RS, Brasil; 2 Universidade Federal do Rio Grande do Sul Porto Alegre RS Brasil Universidade Federal do Rio Grande do Sul, Porto Alegre, RS, Brasil; 3 Universidade Federal de Rio Grande Rio Grande RS Brasil Universidade Federal de Rio Grande, Rio Grande, RS, Brasil

**Keywords:** Noncommunicable diseases, nutritional status, anthropometric parameters, metabolic parameters, polymorphism

## Abstract

**Objective:**

To study associations between polymorphisms in the angiotensin converting enzyme (ACE I/D), actinin 3 (ACTN3 R577X) and paraoxonase 1 (PON1 T(-107)C) genes and chronic diseases (diabetes and hypertension) in women.

**Materials and methods:**

Genomic DNA was extracted from saliva samples of 78 women between 18 and 59 years old used for genetic polymorphism screening. Biochemical data were collected from the medical records in Basic Health Units from Southern Brazil. Questionnaires about food consumption, physical activity level and socioeconomic status were applied.

**Results:**

The XX genotype of ACTN3 was associated with low HDL levels and high triglycerides, total cholesterol and glucose levels. Additionally, high triglycerides and LDL levels were observed in carriers of the TT genotype of PON1, and lower total cholesterol levels were associated to the CC genotype. As expected, women with diabetes/hypertense had increased body weight, BMI (p = 0.02), waist circumference (p = 0.01), body fat percentage, blood pressure (p = 0.02), cholesterol, triglycerides (p = 0.02), and blood glucose (p = 0.01), when compared to the control group.

**Conclusion:**

Both ACTN3 R577X and PON1 T(-107)C polymorphisms are associated with nutritional status and blood glucose and lipid levels in women with diabetes/hypertense. These results contribute to genetic knowledge about predisposition to obesity-related diseases.

## INTRODUCTION

Obesity is an abnormal or excessive fat accumulation that impairs health, and its prevalence has nearly tripled since 1975. It originates from the imbalance between food consumption and energetic expenditure, and is influenced by physiological, genetic and behavioral aspects (1). Worldwide, it is estimated that 38.9% of adults 18 years or older have overweight and 13.1% have obesity, and both prevalence are higher in women (2–4).

In respect to obesity-related comorbidities, type 2 diabetes mellitus (T2D) and hypertension (HAS) are among the most common and dangerous non-communicable diseases (NCD). NCDs cause a large and growing burden of morbidity and mortality and are significant public health challenges worldwide. NCDs develop due to lifestyle, genetic and environmental factors (5). In view of the increased prevalence of NCD, the diagnosis of the population genetic predisposition to these morbidities can be an important tool in the prevention and reduction of treatment costs, through more individual programs and special attention to patients in higher risk.

NCDs are associated to increased blood pressure, dyslipidemia and insulin resistance (6). Peripheral vascular resistance is regulated by angiotensin II and bradykinin; therefore, individuals with the DD genotype for the angiotensin converting enzyme (ACE) gene have increased blood pressure, while further studies are necessary to understand the role of this polymorphism in the development of NCDs (2,3). On the other hand, paraoxonase 1 (PON1) is an enzyme synthesized by the liver (4), with a role in protecting the low-density lipoprotein (LDL) against oxidative changes (5). It has been reported that PON1 activity decreases with diabetes progression (6), in addition to being associated with dyslipidemia (7). The T(-107)C polymorphism has a strong influence on PON1 serum activity, with the CC genotype associated with a higher enzyme activity (8). Alpha-actinin-3 (ACTN3) is a protein found only in fast skeletal muscle fibers (type II), which are responsible for the generation of contractile force in high speed (9). An influence of this R577X polymorphism in ACTN3 has been suggested, with changes in the energetic metabolism (10), which could be potentially involved with diabetes development.

Based on these evidences, the present study aimed to analyze the associations between I/C ACE, R577X ACTN3 and T(-107)C PON1 polymorphisms and metabolic and anthropometric parameters in women with diabetes and hypertension.

## MATERIALS AND METHODS

### Design e population

A case-control study was conducted with 78 women between 18 and 59 years old who attended three Health Clinics in the city of Rio Grande in Southern Brazil. The case group was composed by women with diabetes and/or hypertension. The control group was composed by women who did not have any clinical conditions. The exclusion criteria were pregnancy and history of malignancy. There were not enough male participants to compose a representative sample.

### Ethics, anthropometric aspects and population characteristics

This study was approved by the ethics committee from the Federal University of Pelotas under number 1.552.553, and under the CAAE (Certificate of Presentation for Ethical Appreciation) number 55995216.3.0000.5317. Biochemical data were collected from the medical records in the Health Care Units in Rio Grande.

Anthropometric measurements were performed according to the World Health Organization (WHO) recommendations. The height was measured using a portable stadiometer (WCS Cardiomed^®^) and the body weight (kg), body fat percentage and basal metabolic rate (BMR) (kcal) were measured using a bioelectrical impedance scale (Omron^®^). The body mass index (BMI) was calculated and classified according to the WHO guidelines for adults (≥18 and <60 years old). Individuals were classified as: underweight (BMI <18.5 kg/m²), normal (BMI between 18.5 and 24.9 kg/m²), overweight (BMI between 25.0 and 29.9 kg/m²) and obesity (BMI ≥ 30 kg/m²) (11). Waist circumference was also measured following recommendations, and the cut-off point for adults is defined as less than half the height, according to Brazilian Guidelines (12). For demographic characterization of the population, we applied the questionnaire for socioeconomic level from the Brazilian Association of Research Companies, which classifies individuals into socioeconomic classes A, B1, B2, C1, C2 and D-E. In order to estimate the physical activity level, we used the International Physical Activity Questionnaire short version, which classifies individuals as very active, active, insufficiently active A and B, and sedentary. Finally, to check eating habits, we applied the food consumption questionnaire from the Brazilian Food and Nutrition Surveillance System, which shows food consumption habits considered healthy and unhealthy.

### Sample collection, DNA extraction and genotyping

Samples from the oral cavity were collected with sterile swabs, identified and stored. Genomic DNA extraction was performed following the proteinase K protocol and precipitated with isopropanol, as previously described (13).

For the ACE I/D polymorphism genotyping, we used the following primers: Forward – 5’-CTG GAG ACC ACT CCC ATC CTT TCT-3’ and Reverse – 5’-GAT GTG GCC ATCA CAT TCG TAG A-3’. The PCR reaction was performed using Gotaq (Promega^®,^ cat. No. M7122) in a thermocycler at 95ºC for 5 minutes, 40 cycles with 3 steps – 95 ºC for 10 seconds, 58 ºC for 10 seconds and 72 ºC for 20 seconds - and a final cycle at 72 ºC for 5 minutes. After electrophoresis on 2% agarose gel, alleles deletion (D = 190 bp) and insertion (I = 490 bp) were identified (14). Due to the difficulty to visualize the insertion band, we adapted the protocol and internal primers (hECAri GAT ATG TTT GCA GAC AGT GTA TCA GTG AAG GAA TCG CTT TCC G and hECAfi GCC AAT TCA AGC CCA GTC) were performed in all DD to confirm the genotype or to identify new ID genotypes among these patients.

For the ACTN3 R577X polymorphism genotyping, we used the primers hACTN3f, hACTN3r, hACTN3Tif and hACTN3Cir, as previously described (15). 0.5 µL was used for the first two primers and 0.125 µL and 0.25 µL were used for the last two primers, respectively. PCR reactions were performed using GoTaq^®^ (Promega^®^, cat. No. M7122) at 95 ºC for 2 minutes, 35 cycles of 95 ºC for 10 seconds, 68 ºC for 10 seconds and 72 ºC for 45 seconds, and a final cycle at 72 ºC for 2 minutes. After electrophoresis on 2% agarose gel, it was possible to detect the alleles R with 413 bp and X with 318 bp.

For the PON1 T(-107)C polymorphism genotyping, we used the primers F-5’-AGC TAG CTG CGG ACC CGG CGG GGA GGA G-3’ and R-5’-GGC TGC AGC CCT CAC CAC AAC CC-3’. PCR was performed using Gotaq (Promega^®^, cat. No. M7122) at 93 ºC for 5 minutes, 35 cycles with 3 steps, at 93 ºC, 65 ºC and 72 ºC for 45 seconds each, and a final cycle at 72 ºC for 5 minutes. After this, the product was digested using BrsBi restriction enzyme (New England Biolabs) at 37 ºC for 2 hours. After electrophoresis on 2% agarose gel, we identified the C allele for 28 and 212 bp bands and the T allele for 240 bp bands (16).

### Statistical analysis

The statistical analysis was performed using Stata version 13.0 (Stata Corporation). Data were examined by multivariate analysis of variance. For variables in which the outcome and exposure were dichotomic, we used chi-square. For continuous quantitative outcomes and dichotomic exposure, we used t-test. Finally, for continuous outcomes and ordinal categorical exposure, we used ANOVA. For Bartllet significant (non-homogeneous variances), a non-parametric equivalent test was used: Kruskal-Wallis. The probability value of p < 0.05 was considered statistically significant and a probability between 0.05 and 0.10 was considered as a trend.

## RESULTS

### Characteristics of the study population

The study enrolled 78 women, and the average age for the control group was lower than the diabetes/hypertension group (36.3 ± 11.3 and 48.3 ± 8.3 respectively, p < 0.05). In the diabetes/hypertension group, 5.9% of the women had diabetes, 58.8% had hypertension and 35.5% had both diabetes and hypertension.

Blood pressure (P = 0.02) and blood glucose (P < 0.01) were higher in the diabetes/hypertension group (Table 1). Total cholesterol tended to be higher in the women with diabetes and hypertension (P = 0.09, Table 1). HDL and LDL levels were not significantly different between the groups (Table 1).

**Table 1:** Characteristics of the study population (n = 78) comparing control and diabetes/hypertension groups (mean ± standard deviation)

Characteristics	Control (n = 44)	Diabetes/hypertension (n = 34)	P value
Age (years)	36.3 ± 11.3	48.3 ± 8.3	**<0.01**
Blood pressure (mmHg)	89.8 ± 12.6	97.3 ± 12.4	**0.02**
Total cholesterol (mg/dL)	180.8 ± 42.5	203.8 ± 56.8	0.09
LDL (mg/dL)*	111.8 ± 37.0	128.0 ± 39.5	0.11
HDL (mg/dL)**	48.5 ± 9.8	47.2 ± 9.3	0.61
Triglycerides (mg/dL)	107.9 ± 40.3	177.9 ± 177.4	**0.02**
Blood glucose (mg/dL)	84.6 ± 11.8	135.0 ± 72.8	**<0.01**

* LDL: low density lipoprotein

** HDL: high density lipoprotein.

### Nutritional status

In the nutritional status analysis, higher BMI (P = 0.02) and waist circumference (P = 0.01) values were found, in addition to a trend towards higher fat body percentage (P = 0.08) in women with diabetes and hypertension (Table 2). The consumption of healthy and unhealthy foods was not significantly different between the groups (Table 3).

**Table 2:** Nutritional status of the study population (mean ± standard deviation)

Characteristics	Control (n = 44)	Diabetes/Hypertension (n = 34)	P value
Weight (kg)	70.6 ± 14.6	77.5 ± 17.9	0.07
Height (m)	1.57 ± 0.06	1.56 ± 0.07	0.64
BMI* (kg/m^2^)	28.5 ± 5.3	31.9 ± 7.2	**0.02**
Waist circumference (cm)	92.7 ± 18.5	103.9 ± 18.0	**0.01**
Fat (%)	38.6 ± 8.3	42.1 ± 8.8	0.08
Basal metabolic rate (kcal)	1492.4 ± 270.6	1529.1 ± 222.8	0.53

* BMI: body mass index.

**Table 3:** Consumption of healthy and unhealthy foods by study participants

Food		Control (n = 44)	Diabetes/Hypertension (n = 34)	P value
Considered healthy (%)				
	Bean	30 (68.2)	26 (76.5)	0.42
	Fruits	20 (45.4)	20 (58.8)	0.24
	Vegetables	23 (52.3)	17 (50.0)	0.84
Considered unhealthy (%)				
	Hamburger/sausages	23 (52.3)	12 (35.3)	0.13
	Sugary drinks	32 (72.3)	24 (70.6)	0.83
	Pastas/crackers	16 (36.6)	7 (20.6)	0.13
	Candies	18 (40.9)	16 (47.1)	0.59

### Socioeconomic characteristics and physical activity

The socioeconomic distribution showed to be balanced between the groups and the majority of participants (29.5%) were in the C2 class, with only 3.8% in the B1 and no participants in the A economic class.

No significant difference was observed between the groups for physical activity level, 41% of the total study population was classified as “active”.

### Polymorphisms distribution and frequencies

Table 4 shows the genotype distribution for each polymorphism studied. The data were subjected to Hardy-Weinberg equilibrium analysis. The ACE and ACTN3 genotypes show to be balanced, whereas PON1 showed an imbalance in terms of Hardy-Weinberg in both groups.

**Table 4:** Distribution and Hardy-Weinberg equilibrium of ACE, PON1 e ACTN3 genotypes

Genotype	Control			Diabetes/Hypertension			All			Fisher
	N	%		n	%		n	%		P value
**ACE**										
DD	24	54.5		13	38.2		37	47.4		0.18
ID	16	36.4		13	38.2		29	37.2		1.00
II	4	9.1		8	23.5		12	15.4		0.11
HWE p value	0.31			0.2			0.13			
Allele I frequency		0.27			0.43			0.34		
**PON1**										
TT	4	9.1		7	20.6		11	14.1		0.19
CT	33	75.0		23	67.6		56	71.8		0.61
CC	7	15.9		4	11.8		11	14.1		0.75
HWE p value	<0.001			0.03			<0.001			
Allele C frequency		0.53			0.46			0.50		
**ACTN3**										
RR	15	34.1		9	26.5		24	30.8		0.62
RX	19	43.2		12	35.3		31	39.7		0.64
XX	10	22.7		13	38.2		23	29.5		0.21
HWE p value	0.41			0.13			0.07			
Allele X frequency		0.44			0.55			0.49		

### Association of metabolic and anthropometric data with genotype

Table 5 shows sample characteristics and the distribution of ACE (I/D), PON1 T(-107)C and ACTN3 R577X polymorphisms.

**Table 5:** Sample characteristics and relationship with the genotypes

Characteristics	ACE (I/D)				P value	PON1 (C-(107)T)			P value	ACTN3 (R577X)			P value
	DD	ID		II		TT	CT	CC		RR	RX	XX	
Age	41.2 ± 11.8	40.6 ± 11.2		44.8 ± 13.1	0.60	46.5 ± 6.7	41.4 ± 12.9	37.4 ± 7.6	0.18	37.3 ±11.2	41.8 ± 11.7	45.6 ± 11.3	0.05
Weight	72.8 ± 16.4	76.0 ± 18.1		70.8 ± 12.0	0.60	77.9 ± 18.5	73.6 ± 14.9	69.9 ± 21.7	0.54	72.2 ± 17.3	73.1 ± 12.6	76.0 ± 20.2	0.73
Height	1.56 ± 0.08	1.56 ± 0.05		1.54 ± 0.06	0.98	1.53 ± 0.06	1.57 ± 0.07	1.56 ± 0.07	0.23	1.57 ± 0.06	1.57 ±0.08	1.54 ± 0.05	0.34
BMI*	29.6 ± 6.8	31.1 ± 6.8		28.9 ± 4.3	0.53	33.6 ± 8.4	29.7 ± 5.4	28.4 ± 8.4	0.10	29.3 ± 6.6	29.3 ± 4.2	31.9 ± 8.5	0.74
WC*	96.4 ± 23.4	100.6 ± 16.0		96.0 ± 9.2	0.63	103.8 ± 20.1	97.2 ± 19.1	95.3 ± 18.4	0.55	96.7 ± 15.6	95.2 ± 21.4	102.7 ± 19.1	0.37
Fat %*	39.5 ± 9.7	41.5 ± 8.0		39.4 ± 7.2	0.63	41.9 ± 11.8	40.2 ± 7.7	38.8 ± 10.3	0.73	39.1 ± 8.6	40.3 ± 8.11	41.3 ± 9.7	0.69
BMR*	1502.2 ± 239.2	1533.2 ± 263.5		1477.2 ± 259.8	0.80	1625.2 ± 284.2	1475.1 ± 211.9	1562 ± 346.3	0.16	1497.1 ± 242.0	1494.0 ± 246.4	1542.9 ± 267.8	0.77
Mean pressure	92.0 ± 11.3	93.9 ± 12.1		95.3 ± 18.8	0.72	94.0 ± 11.8	94.1 ± 13.8	88.3 ± 9.2	0.44	97.8 ± 15.2	91.2 ± 12.0	90.7 ± 10.4	0.12
Total cholesterol	192.5 ± 49.5	196.3 ± 39.6		187.2 ± 74.2	0.90	193.8 ± 65.2	199.7 ± 50.1	161.7 ± 22.5	0.04	173.1 ± 33.7	187.0 ± 60.3	216.8 ± 45.5	0.03
LDL*	118.7 ± 40.9	124.4 ± 31.3		116.6 ± 47.6	0.84	130.6 ± 22.3	122.9 ± 43.6	95.7 ± 22.1	0.03	107.1 ± 27.5	122.3 ± 42.3	129.4 ± 41.9	0.22
HDL*	47.3 ± 10.2	48.5 ± 9.1	47.6 ± 9.1		0.92	46.0 ± 10.2	48.8 ± 9.5	45.8 ± 9.2	0.55	47.0 ± 10.0	52.0 ± 6.7	43.6 ± 10.3	0.01
Triglycerides	132.9 ± 77.3	122.8 ± 44.3	209.3 ± 277.8		0.71	180.5 ± 95.3	144.0 ± 155.3	99.9 ± 39.3	0.04	100.5 ± 41.5	113.2 ± 42.2	208.9 ± 211.2	<0.05
Blood glucose	122.8 ± 78.0	97.2 ± 25.4	98.3 ± 19.0		0.74	134.8 ± 73.7	98.0 ± 31.9	131.4 ± 103.1	0.18	104.2 ± 74.7	93.6 ± 18.0	134.7 ± 64.7	<0.05

* BMI: body mass index; WC: waist circumference; Fat %: fat perceptual; BMR: basal metabolic rate; LDL: low density lipoprotein; HDL: high density lipoprotein.

A lower age was observed for the group RR genotype of ACTN3 R755X polymorphism (RR -> 37.3 ± 11.2 years; RX -> 41.8 ± 11.7 XX -> 45.6 ± 11.3; p = 0.05). No differences were observed for weight or height in all genotypes studied.

It was also not possible to show differences in the nutritional status according to BMI, waist circumference and fat percentage, and also no differences in socioeconomic and physical activity data compared to genotypes.

**Figure 1:**
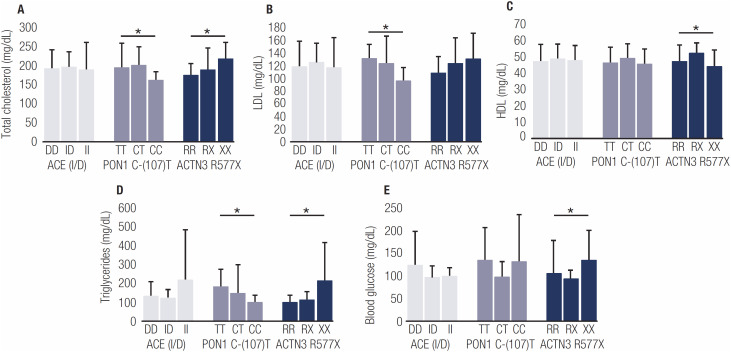
Changes in blood parameters compared to ACE (I/D), PON1 C-(107)T and ACTN3 R577X polymorphisms. (**A**) Total blood cholesterol, (**B**) low density lipoprotein (LDL), (**C**) high density lipoprotein (HDL), (**D**) triglycerides, and (E) blood glucose levels. *P < 0.05

The XX genotype of ACTN3 R577X was associated with higher blood glucose, total cholesterol and triglyce­rides, when compared to other genotypes. A relationship between this genotype and lower HDL values was observed (Figure 1). On the PON1 polymorphism, the CC genotype showed lower total cholesterol, LDL and triglycerides values. A trend towards higher BMI was found in the TT genotype. No association was found between the ACE I/D polymorphism genotypes and the studied characteristic (Figure 1).

## DISCUSSION

This study analyzed the association of genetic polymorphisms with nutritional status and the risk of developing diabetes and hypertension in women. We showed that the TT genotype of PON1 T(-107)C polymorphism and the XX genotype of ACTN3 R577X polymorphism are associated with higher triglycerides levels and the XX genotype is associated with higher blood glucose and total cholesterol levels and lower HDL levels in the studied population.

Regarding the ACTN3 R577X polymorphism, the XX genotype was associated with higher blood glucose, triglycerides and total cholesterol values, and lower HDL levels. This relationship between ACTN3 and glucose and lipid metabolism has been suggested to be a result of changes in blood pressure regulation in previous studies (17,18). Although this polymorphism has been more studied for athletic performance, mainly in respect to sprint/endurance talent, our group, among others, has shown that the absence of a functional ACTN3 protein is associated with metabolic changes and also with different responses to diet intervention focused on dyslipidemia control (13). A study in Western Mexico has found that the ACTN3 R577X polymorphism was associated with higher glucose and triglyceride levels in woman (19). This was the reason why we included this polymorphism in the present study. Further studies are necessary to fully understand this polymorphism and metabolic changes, and which mechanism is involved in these changes.

ACE is known to connect the renin-angiotensin and the kallikrein-kinin systems. This enzyme is responsible for producing angiotensin II, a strong vasoconstrictor, from angiotensin I, and degrade the bradykinin peptide, thus decreasing the levels of this vasodilator. The DD genotype is associated with high ACE serum activity, therefore, this genotype could be associated with high blood pressure (11,20). Although some authors indicate a positive correlation between DD genotype of ACE I/D polymorphism and hypertension (21), this study did not find this association. These results corroborate the study by Mondry and cols. (22), in which this polymorphism did not contribute for HAS prevalence and severity. In addition, another study (23) did not find any differences between groups of wemen with normal pressure and hypertension regarding the frequency of I/D polymorphism.

The CC genotype of the PON1 T(-107)C polymorphism was associated with lower triglycerides, total cholesterol and LDL levels. These findings are in line with Abbott and cols. (24) and Ikeda and cols. (25). The CC genotype is associated with higher PON1 serum activity (4). Low levels of PON1 lead to increased endothelial damage and, consequently, increased risk of coronary disease (26). PON1 plays an antioxidant role in lipid metabolism, and these actions of PON1 can exert a protective effect on the progression of atherosclerosis and cardiovascular disease (27). The lack of balance in terms of Hardy-Weinberg for PON1 polymorphisms could be related to a selection bias, since this genotyping is part of our laboratory routine and the genotype was double-checked for each patient. In our experience with this polymorphism, the problem with HWE happens mainly in small sample size. Genotyping performed in other cities have not presented this type of bias, which may be related to a low number of participants in the present study. This low sample size and the excess of heterozygotes results for PON1 SNP reduced your power to detect more significant differences in others parameters. Despite it, it was possible to see effects of CC genotype in triglycerides and cholesterol levels profiles.

Overall, the BMI values in both groups were above those recommended by WHO, and the overall average in the study population was 29.2 kg/m². The anthropometric and body composition data (BMI, weight, fat perceptual and waist circumference) were higher in the group of women with diabetes and hypertension, further confirming the association between overweight/obesity and an increased risk of developing diabetes and HAS. This is in line with the reported by Patel and cols. (28), which showed a positive relationship between overweight/obesity and patients with hypertension, diabetes or both. The age difference between the groups could be a bias and was expected, since it is widely described in the literature that risk factors for these diseases are more frequent with advancing age (29,30). Regarding diabetes, Collins and cols. (31) mention age as the most common risk factor. Structural changes with advancing age also favor increases in blood pressure (32). In the present study, the mean blood pressure was higher in women with diabetes and hypertension, however, it was within the normal range in both groups (<100 mmHg). This may be related to the use of antihypertensive drugs. However, the different distribution of the studied alleles and their association with the symptoms contribute with the knowledge in the area.

Additionally, it is important to mention the limitations of the present study. The external replication of the study is limited, in particular for variants that were not previously reported to be associated with diabetes and hypertension, like ACTN3. Only nominal significant p-values have been met. None adjustment to multiple hypotheses that have been tested. Therefore, we cannot exclude the possibility of a chance finding.

In conclusion, in our study the XX genotype of the ACTN3 R577X polymorphism is associated to higher triglycerides and fasting glucose concentrations in women with diabetes and hypertension. Additionally, higher triglycerides levels were observed for the TT genotype of PON1 T(-107)C polymorphism. Although the genotype was not directly linked to the disease incidence itself, it has an effect on important parameters that predispose women to diabetes and hypertension. These results contribute to genetic information about predisposition to obesity-related diseases.
